# Supplementary material for: The development of Nanosota-1 as anti-SARS-CoV-2 nanobody drug candidates
Source: eLife. 2021 Aug 2;10:e64815. doi: 10.7554/eLife.64815 (PMC8354634; doi:10.7554/eLife.64815)
Supplement: Figure 2—figure supplement 4—source data 1. [file elife-64815-fig2-figsupp4-data1.zip › Figure 2-figure supplement 4-source data 1/SDS-PAGE gels and blots.docx]

**Source files for “Binding interactions between *Nanosota-1* and SARS-CoV-2 RBD” (related to Figure 2-figure supplement 3)**

This zip archive contains all gels and blots shown in Figure 2-figure supplement 3. Both the original files of the full raw unedited gels or blots and figures with the uncropped gels or blots with the relevant bands labelled are included. The filename of each source data indicates which panel of the figure it is related to.
