# Supplementary figures and images for: The development of Nanosota-1 as anti-SARS-CoV-2 nanobody drug candidates
Source: eLife. 2021 Aug 2;10:e64815. doi: 10.7554/eLife.64815 (PMC8354634; doi:10.7554/eLife.64815)

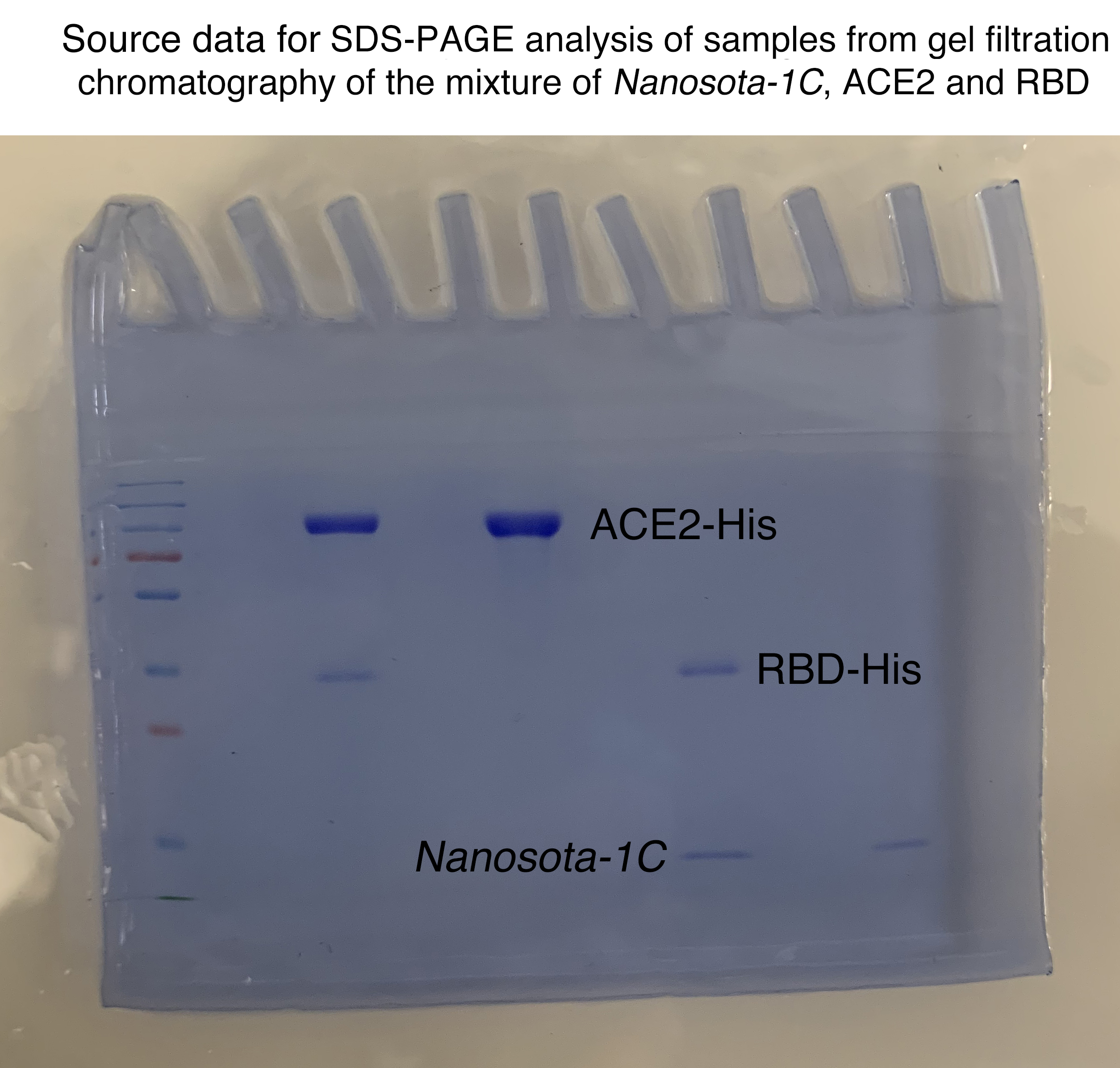

Supplement: Figure 2—figure supplement 4—source data 1. [file elife-64815-fig2-figsupp4-data1.zip › Figure 2-figure supplement 4-source data 1/Figure 2-figure supplement 4C-source data 4 labeled.tif]

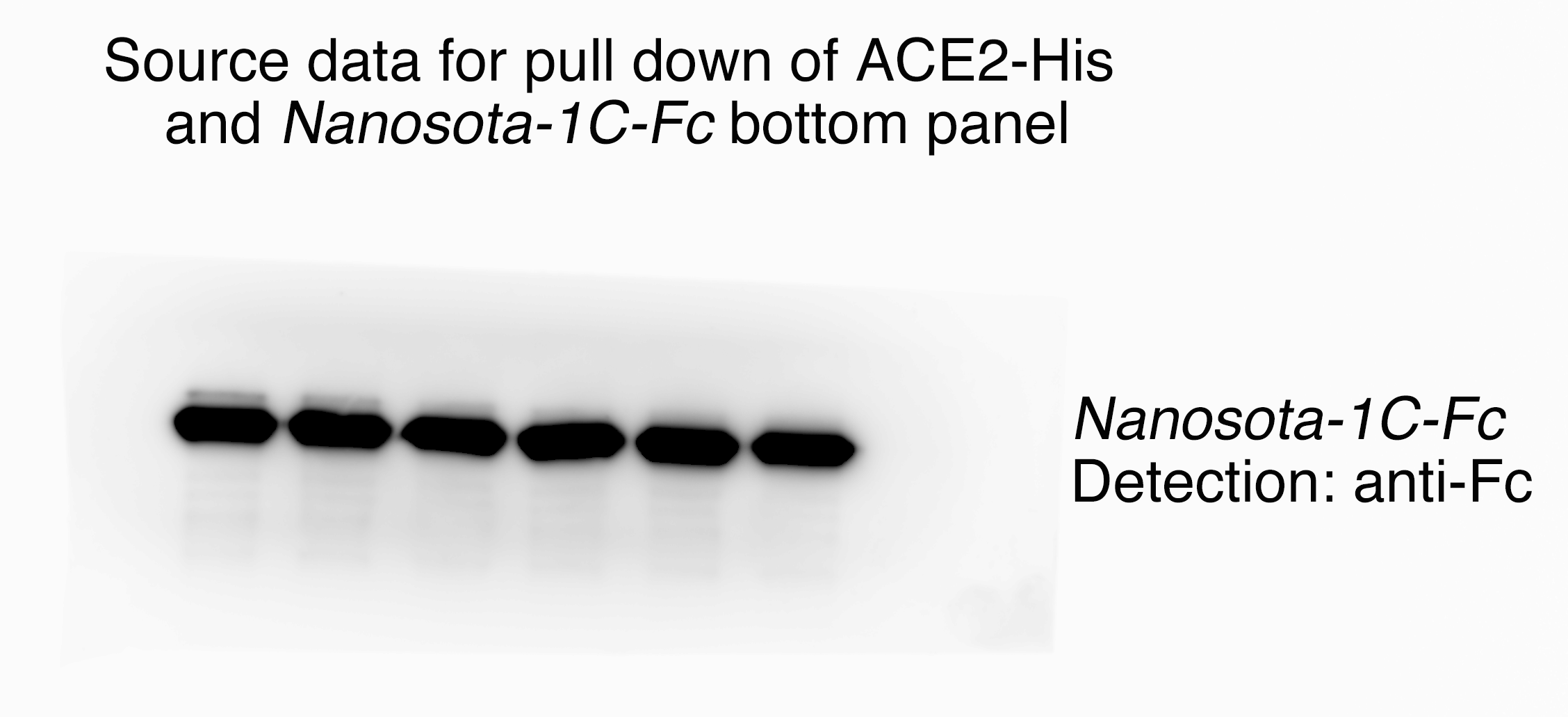

Supplement: Figure 2—figure supplement 4—source data 1. [file elife-64815-fig2-figsupp4-data1.zip › Figure 2-figure supplement 4-source data 1/Figure 2-figure supplement 4B-source data 3 labeled.tif]

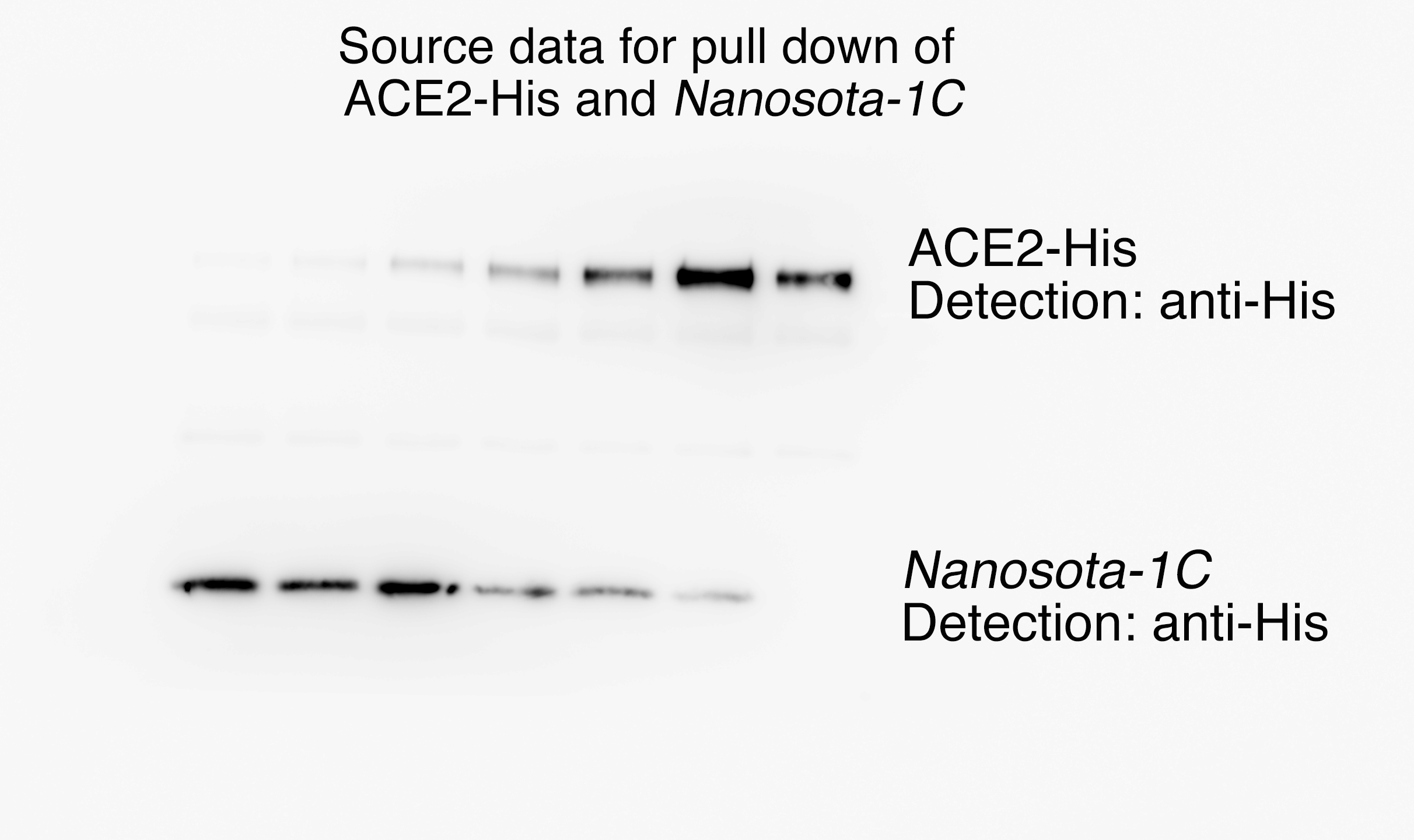

Supplement: Figure 2—figure supplement 4—source data 1. [file elife-64815-fig2-figsupp4-data1.zip › Figure 2-figure supplement 4-source data 1/Figure 2-figure supplement 4A-source data 1 labeled.tif]

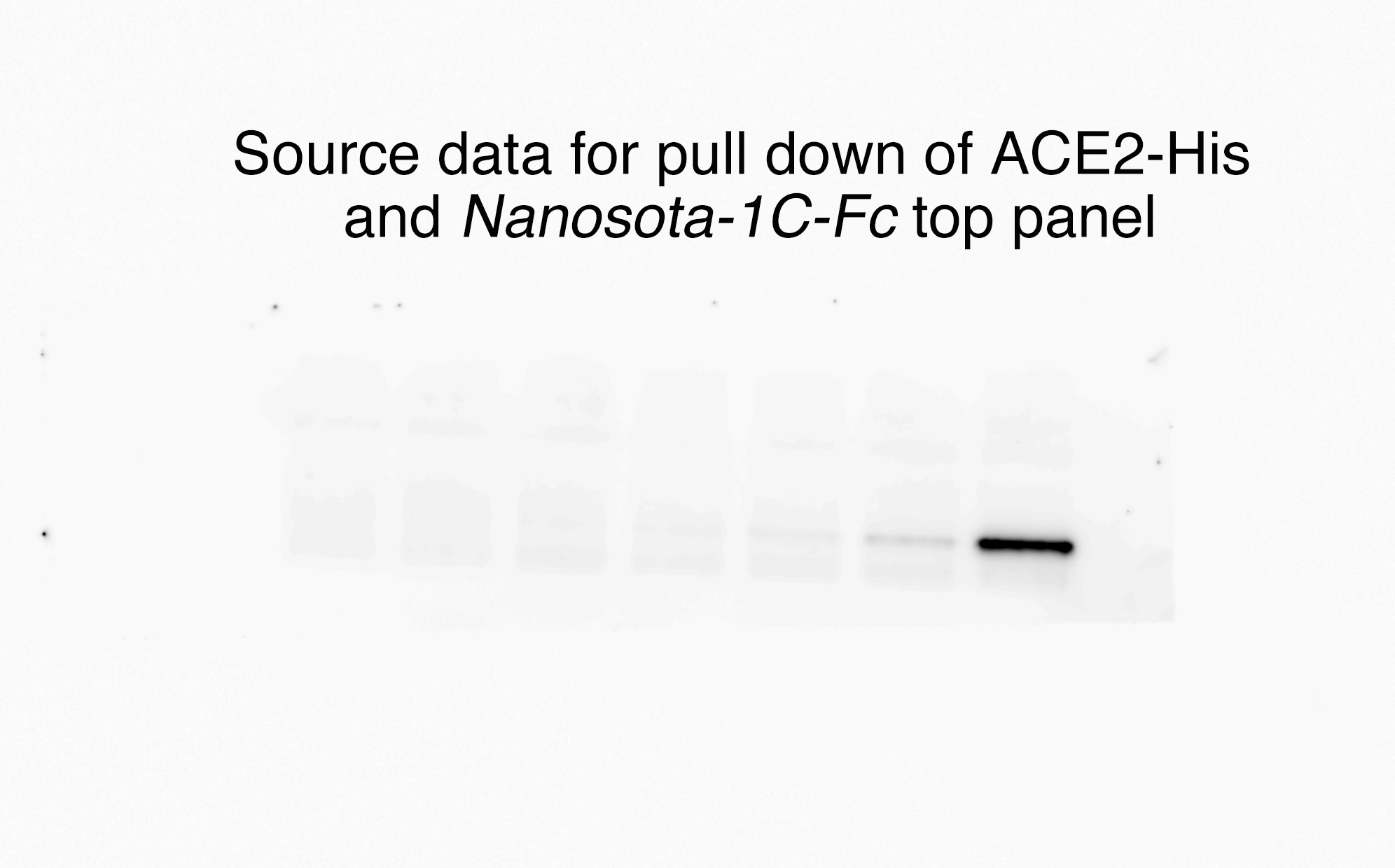

Supplement: Figure 2—figure supplement 4—source data 1. [file elife-64815-fig2-figsupp4-data1.zip › Figure 2-figure supplement 4-source data 1/Figure 2-figure supplement 4B-source data 2.tif]

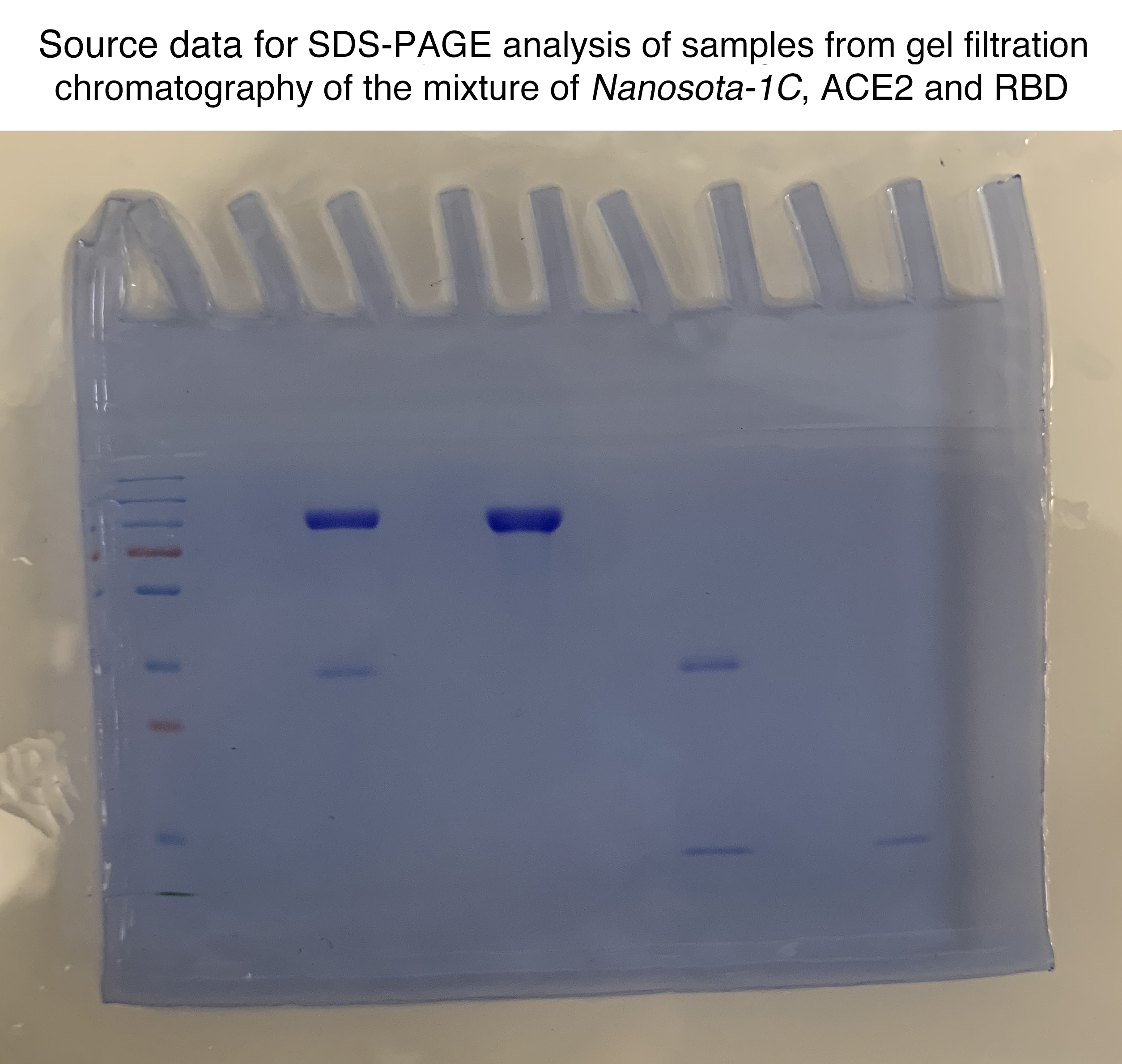

Supplement: Figure 2—figure supplement 4—source data 1. [file elife-64815-fig2-figsupp4-data1.zip › Figure 2-figure supplement 4-source data 1/Figure 2-figure supplement 4C-source data 4.tif]

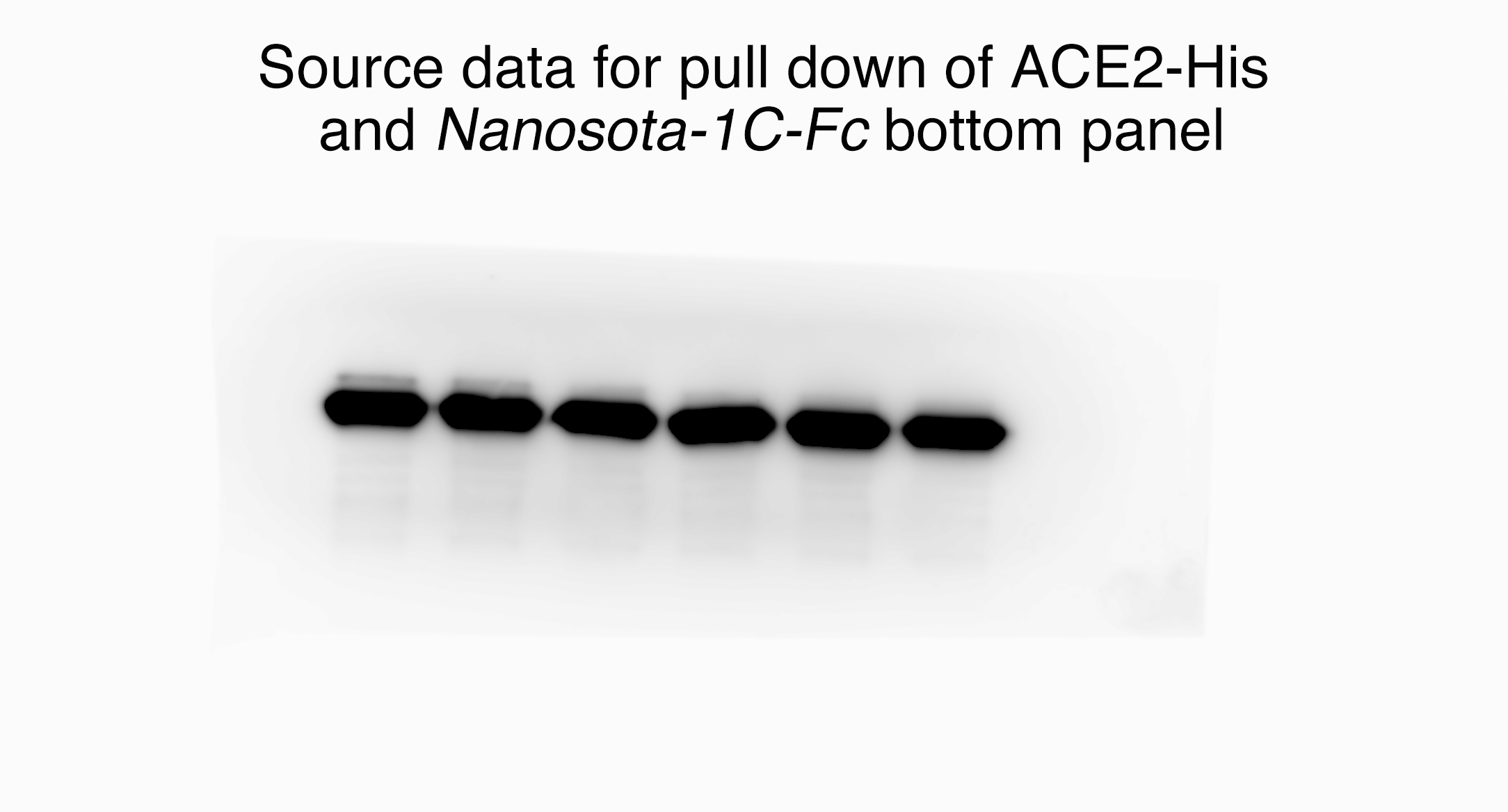

Supplement: Figure 2—figure supplement 4—source data 1. [file elife-64815-fig2-figsupp4-data1.zip › Figure 2-figure supplement 4-source data 1/Figure 2-figure supplement 4B-source data 3.tif]

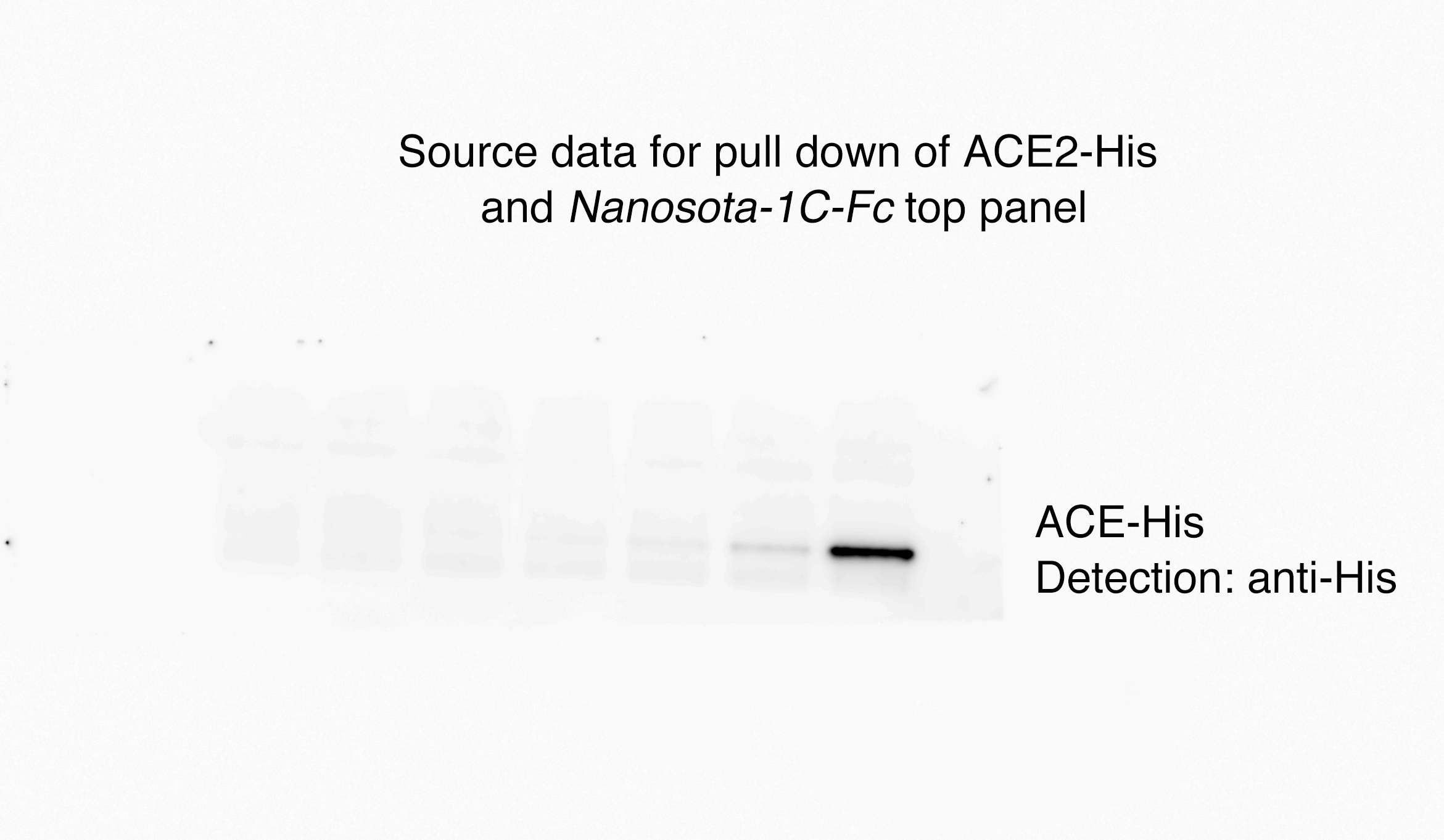

Supplement: Figure 2—figure supplement 4—source data 1. [file elife-64815-fig2-figsupp4-data1.zip › Figure 2-figure supplement 4-source data 1/Figure 2-figure supplement 4B-source data 2 labeled.tif]

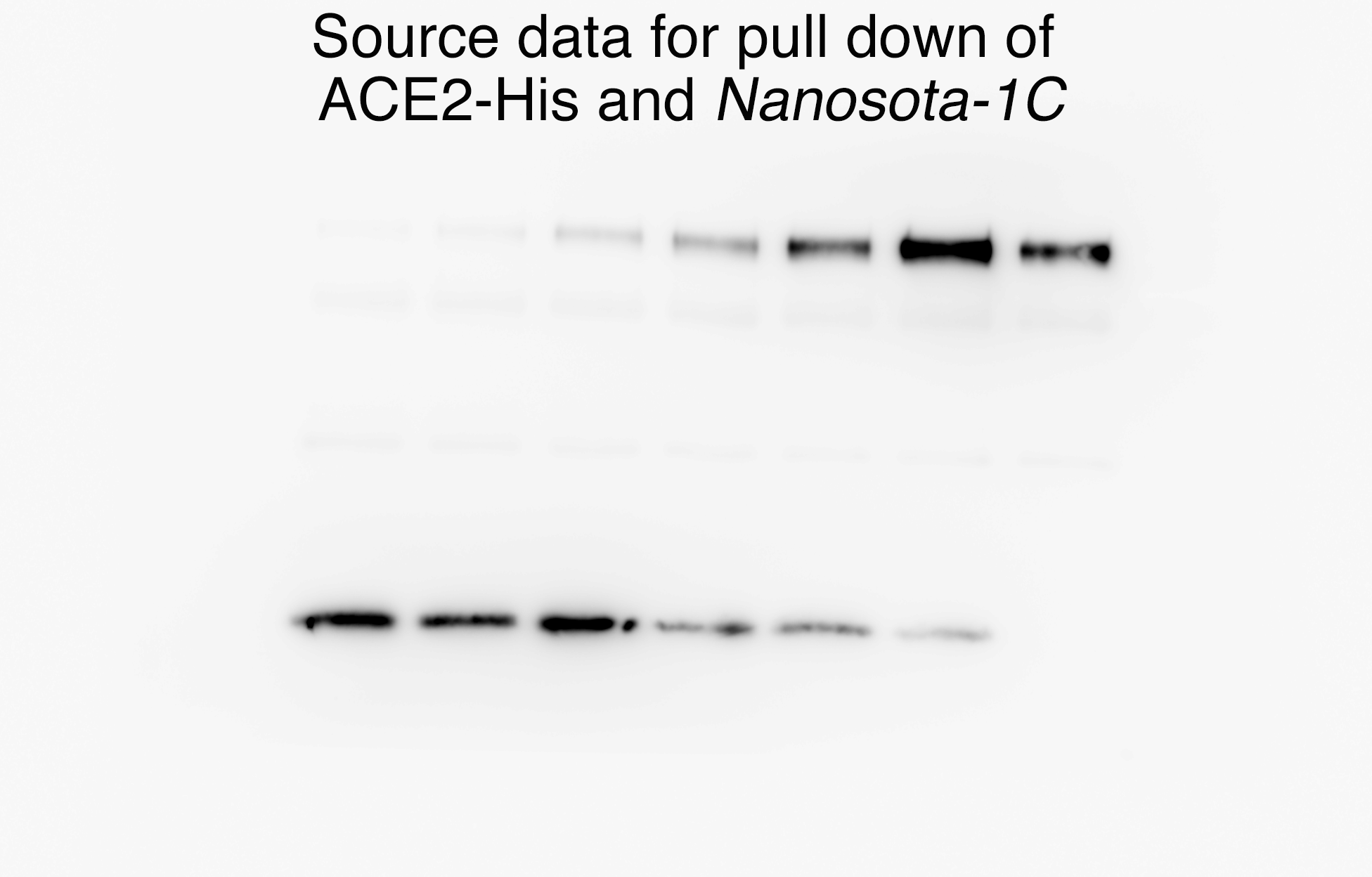

Supplement: Figure 2—figure supplement 4—source data 1. [file elife-64815-fig2-figsupp4-data1.zip › Figure 2-figure supplement 4-source data 1/Figure 2-figure supplement 4A-source data 1.tif]

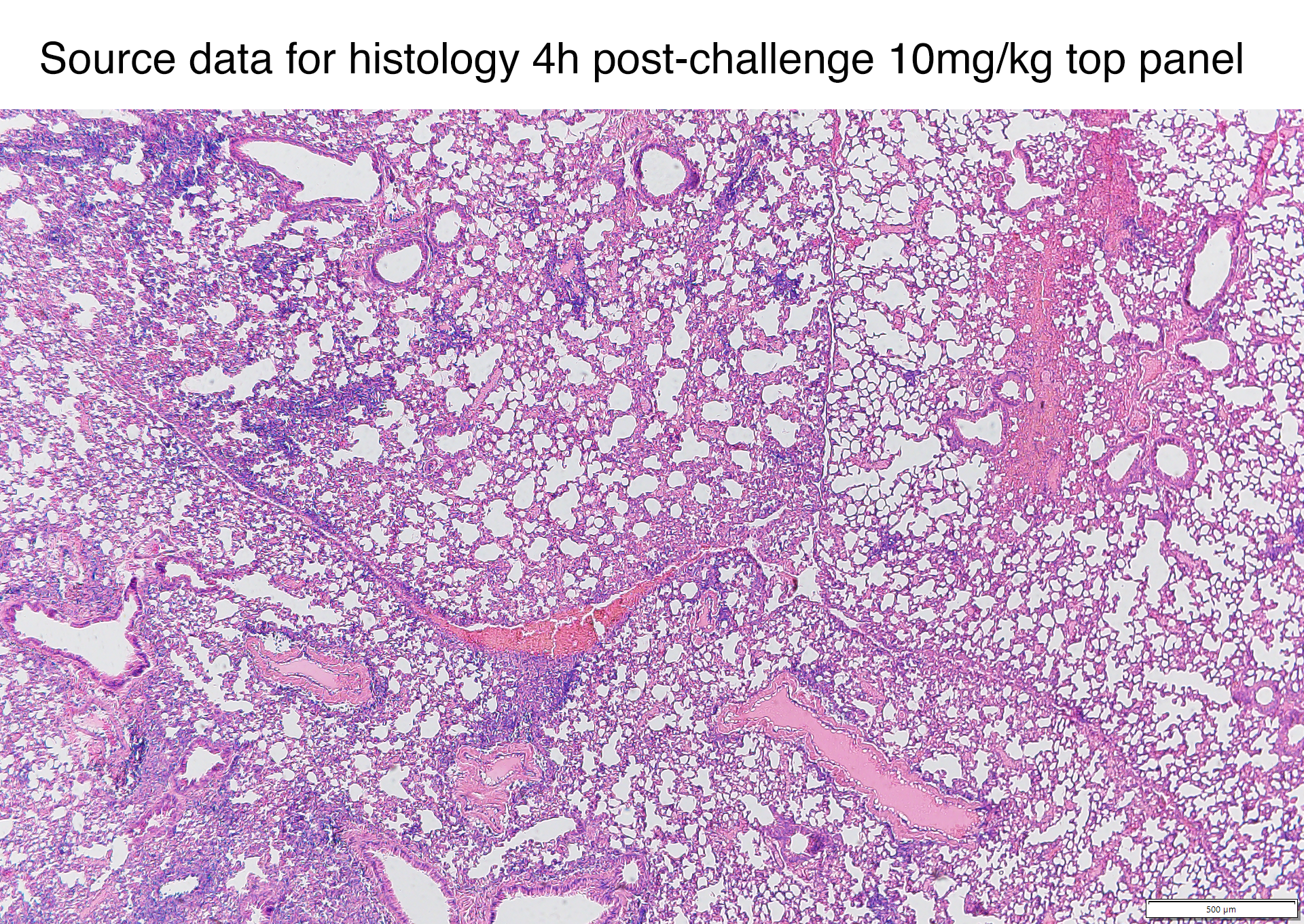

Supplement: Figure 4—source data 1. [file elife-64815-fig4-data1.zip › Figure 4D-source data 1/Figure 4D-source data 9.tif]

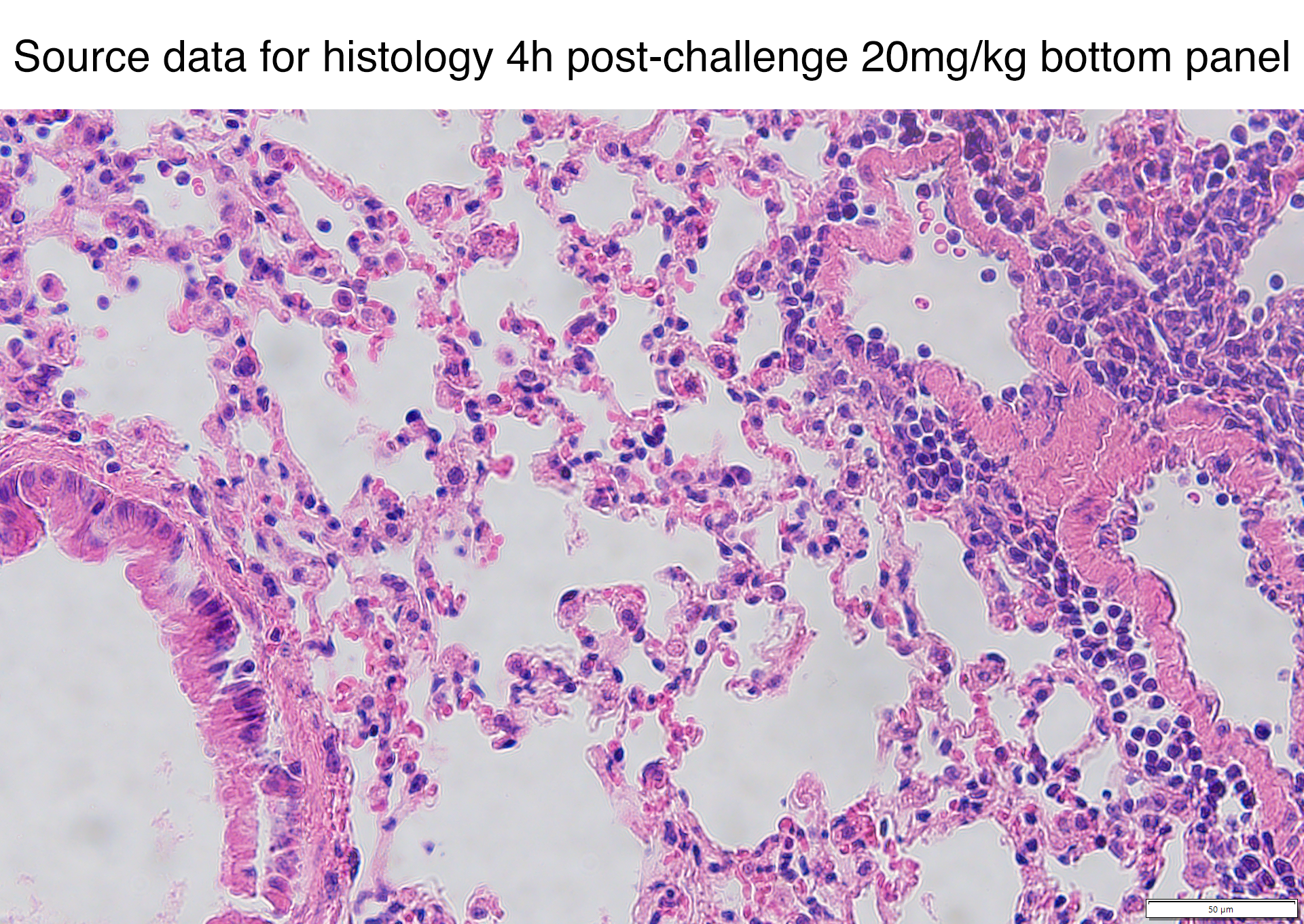

Supplement: Figure 4—source data 1. [file elife-64815-fig4-data1.zip › Figure 4D-source data 1/Figure 4D-source data 8.tif]

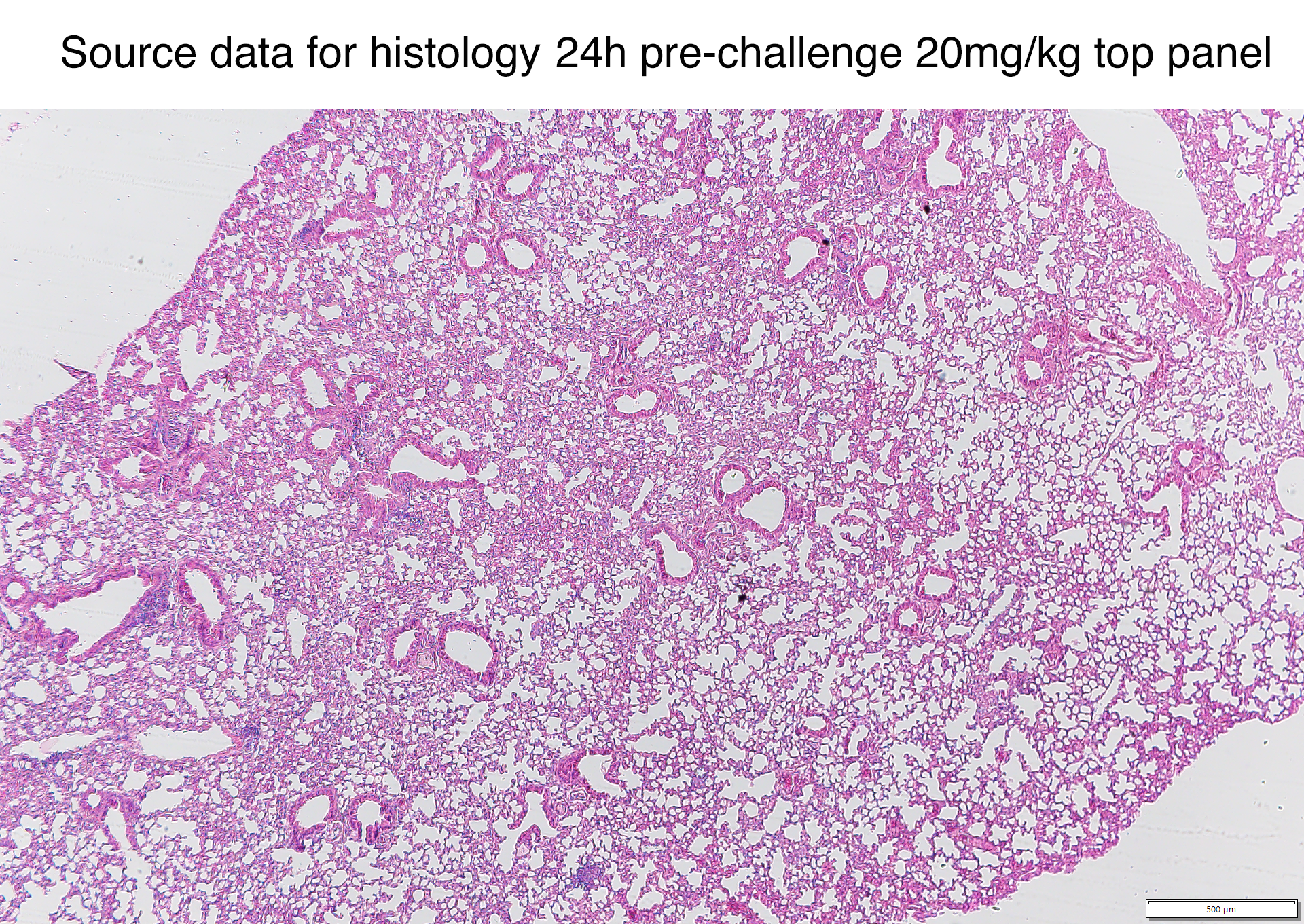

Supplement: Figure 4—source data 1. [file elife-64815-fig4-data1.zip › Figure 4D-source data 1/Figure 4D-source data 3.tif]

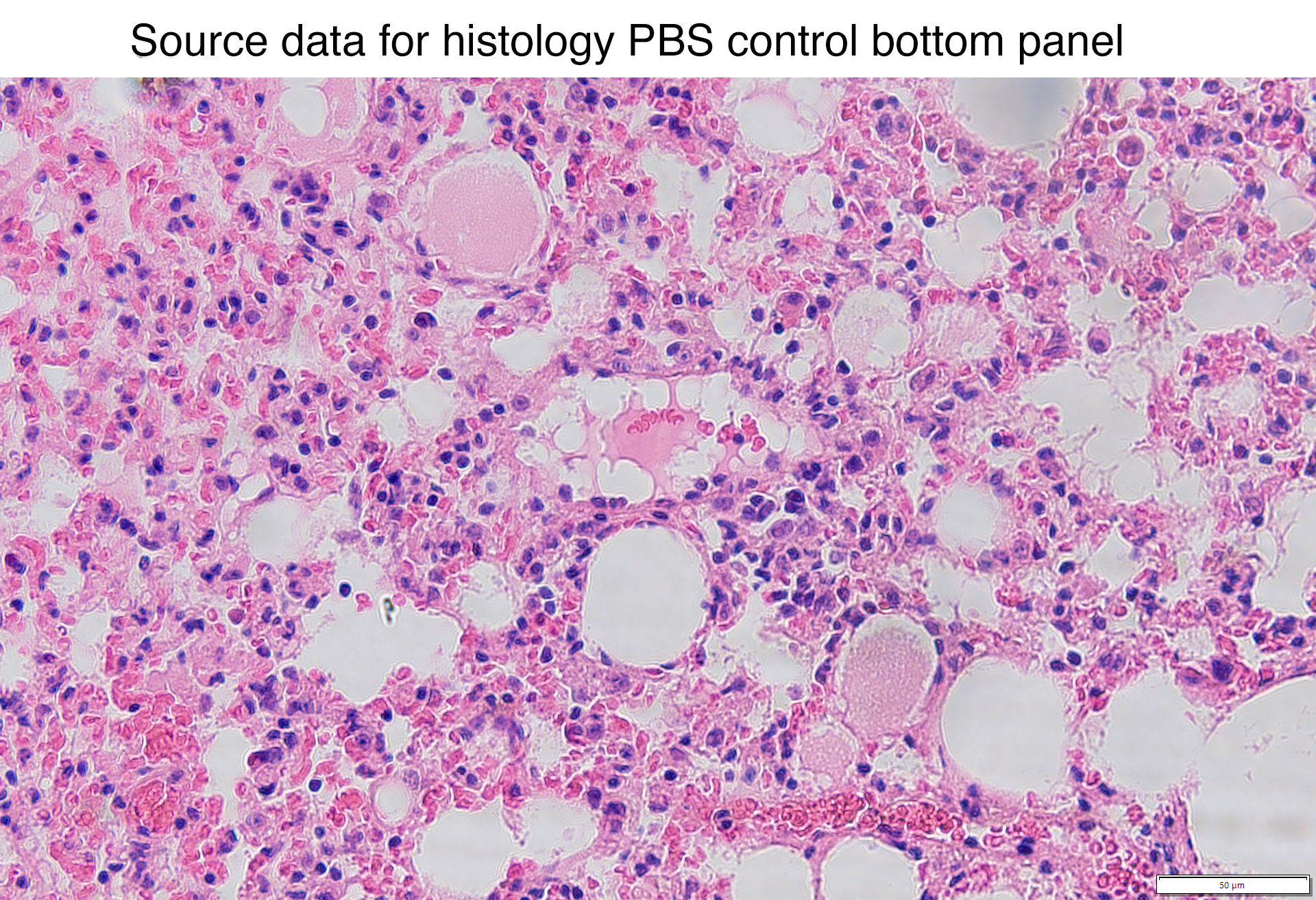

Supplement: Figure 4—source data 1. [file elife-64815-fig4-data1.zip › Figure 4D-source data 1/Figure 4D-source data 2.tif]

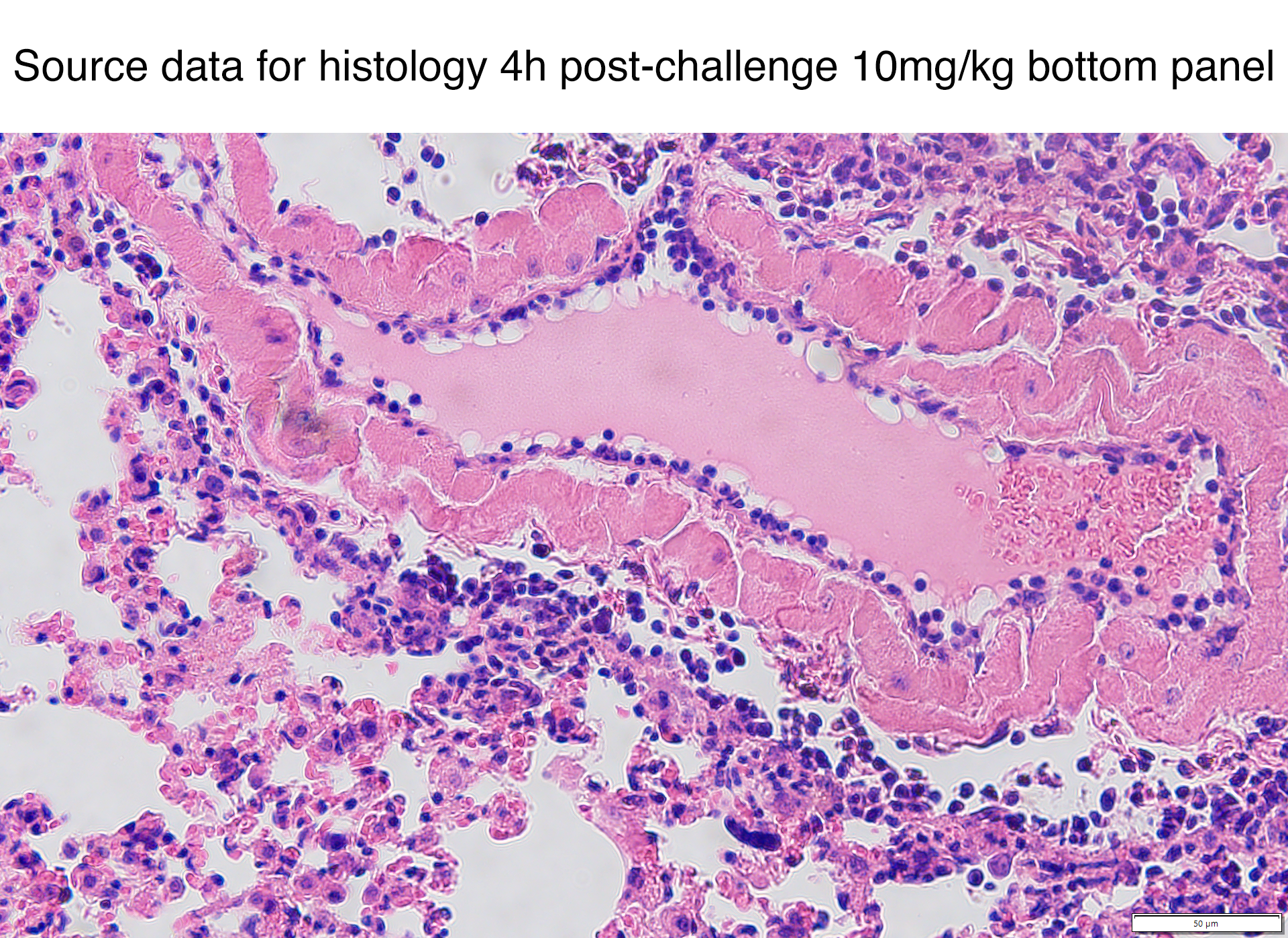

Supplement: Figure 4—source data 1. [file elife-64815-fig4-data1.zip › Figure 4D-source data 1/Figure 4D-source data 10.tif]

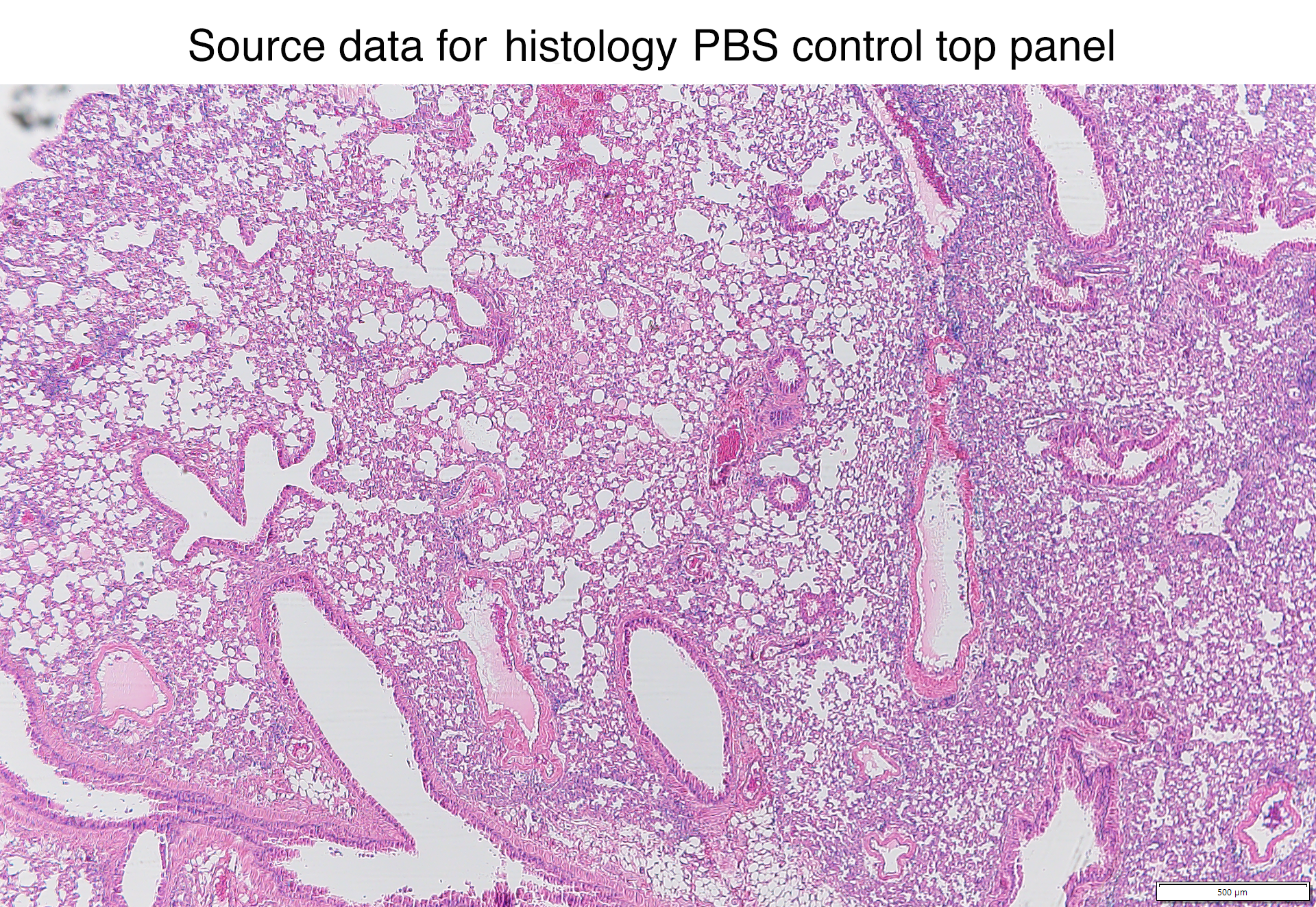

Supplement: Figure 4—source data 1. [file elife-64815-fig4-data1.zip › Figure 4D-source data 1/Figure 4D-source data 1.tif]

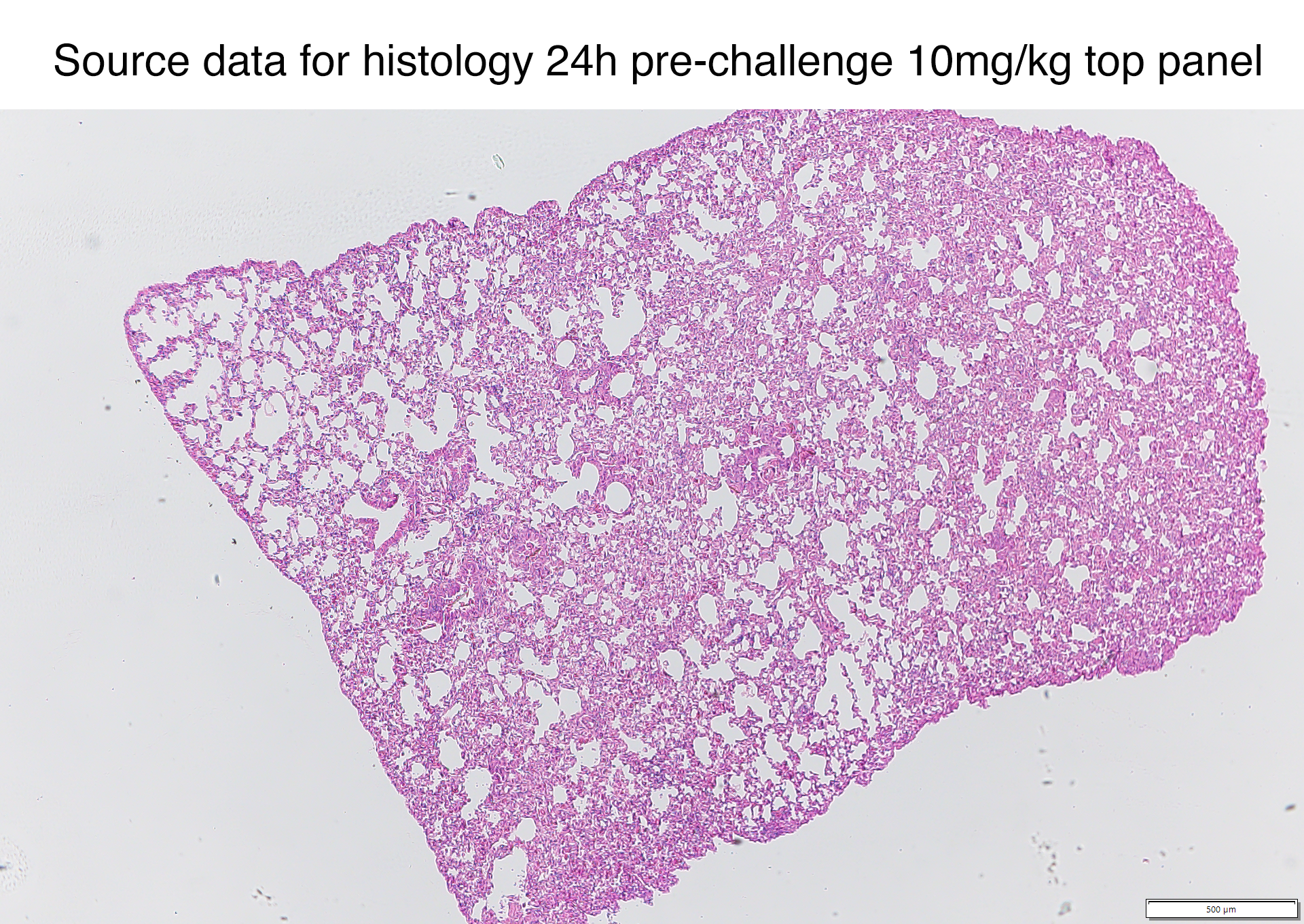

Supplement: Figure 4—source data 1. [file elife-64815-fig4-data1.zip › Figure 4D-source data 1/Figure 4D-source data 5.tif]

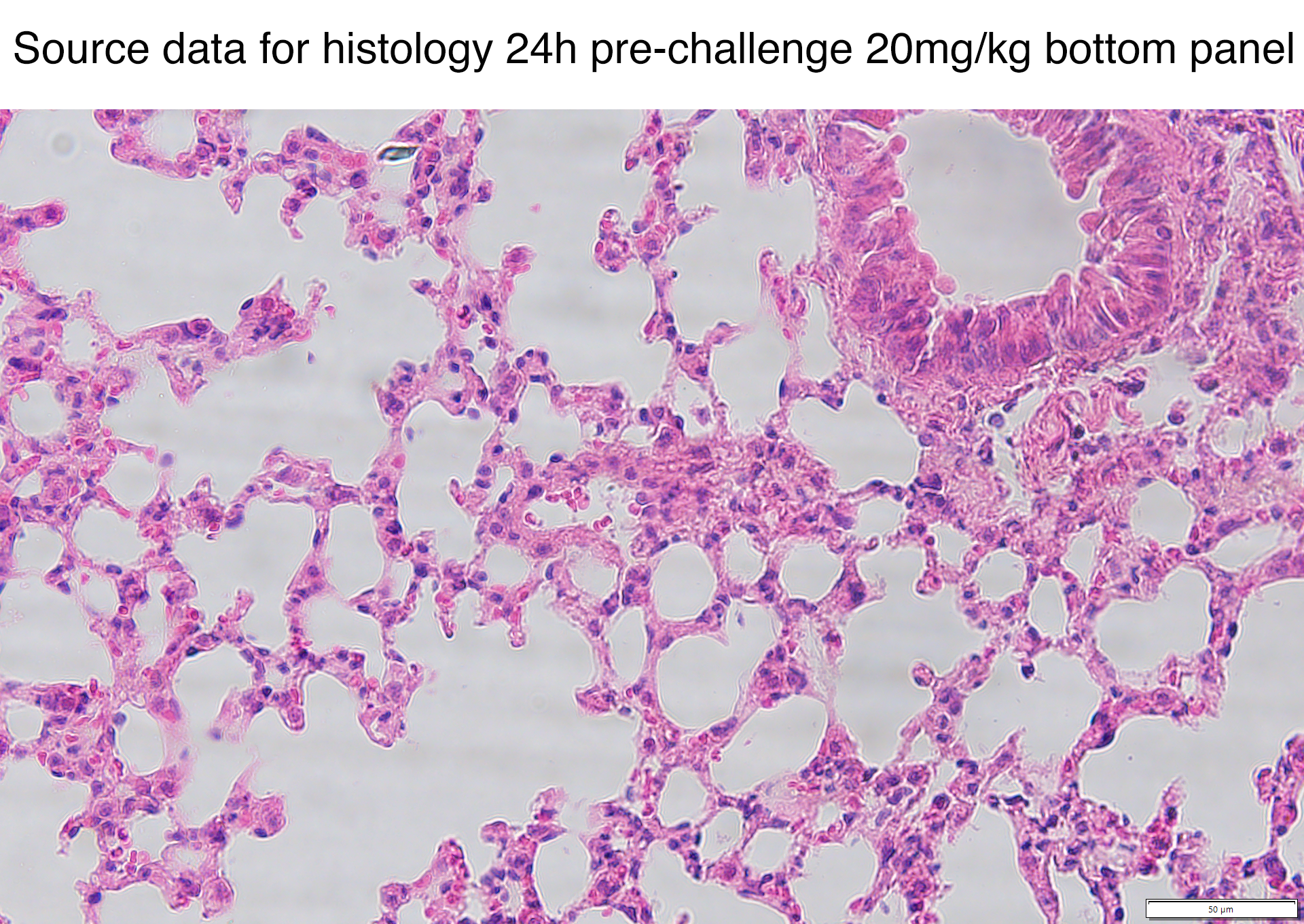

Supplement: Figure 4—source data 1. [file elife-64815-fig4-data1.zip › Figure 4D-source data 1/Figure 4D-source data 4.tif]

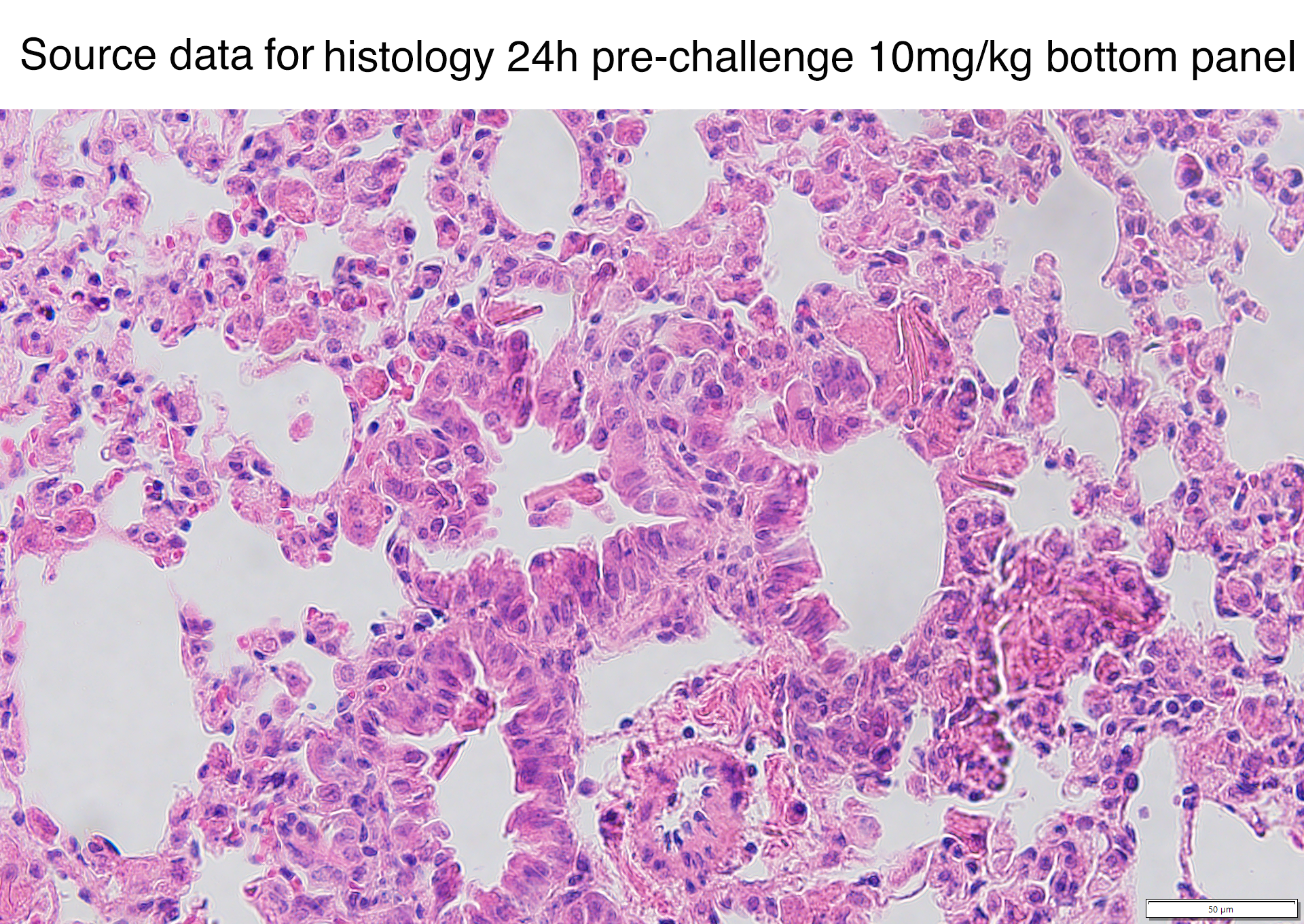

Supplement: Figure 4—source data 1. [file elife-64815-fig4-data1.zip › Figure 4D-source data 1/Figure 4D-source data 6.tif]

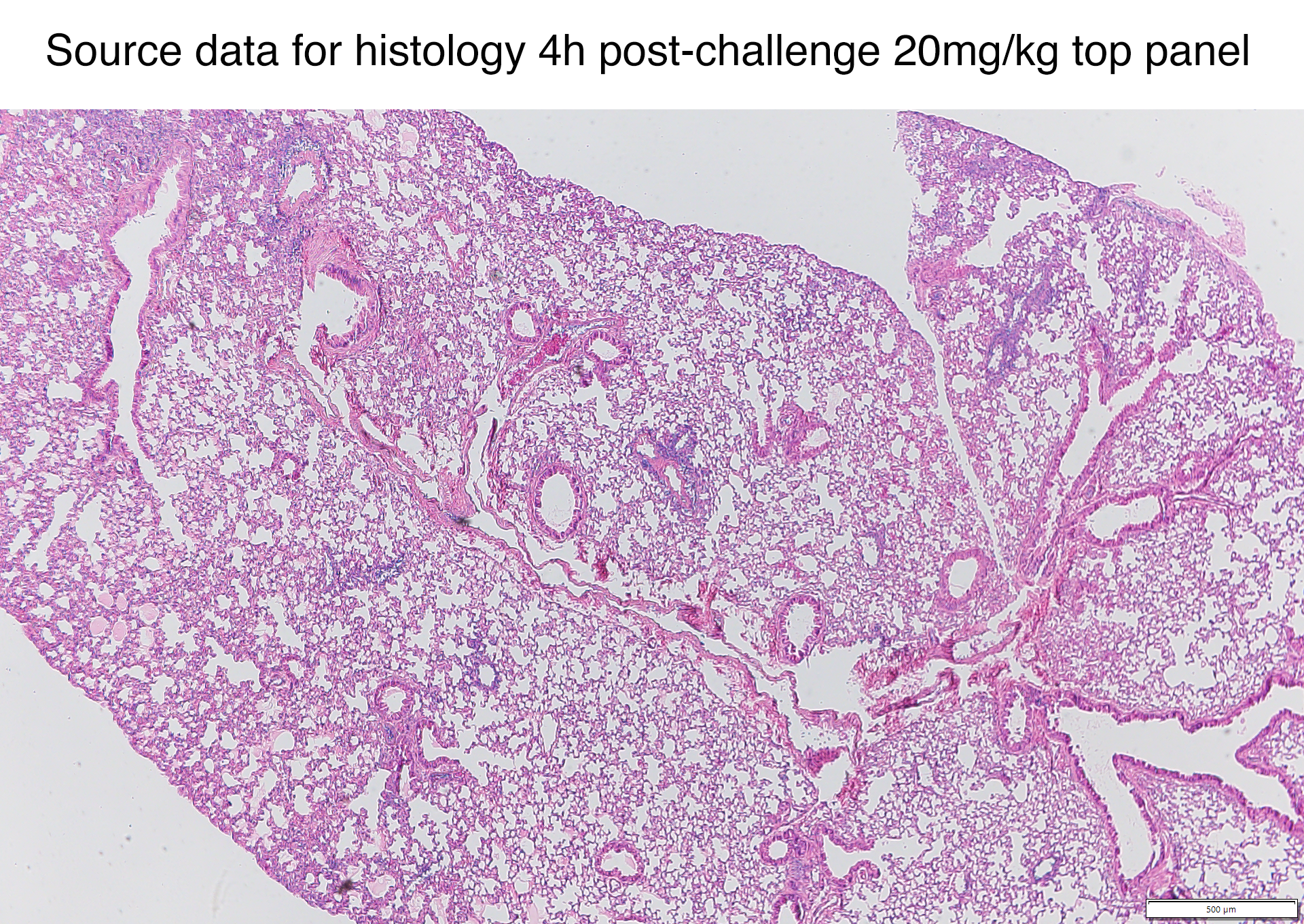

Supplement: Figure 4—source data 1. [file elife-64815-fig4-data1.zip › Figure 4D-source data 1/Figure 4D-source data 7.tif]

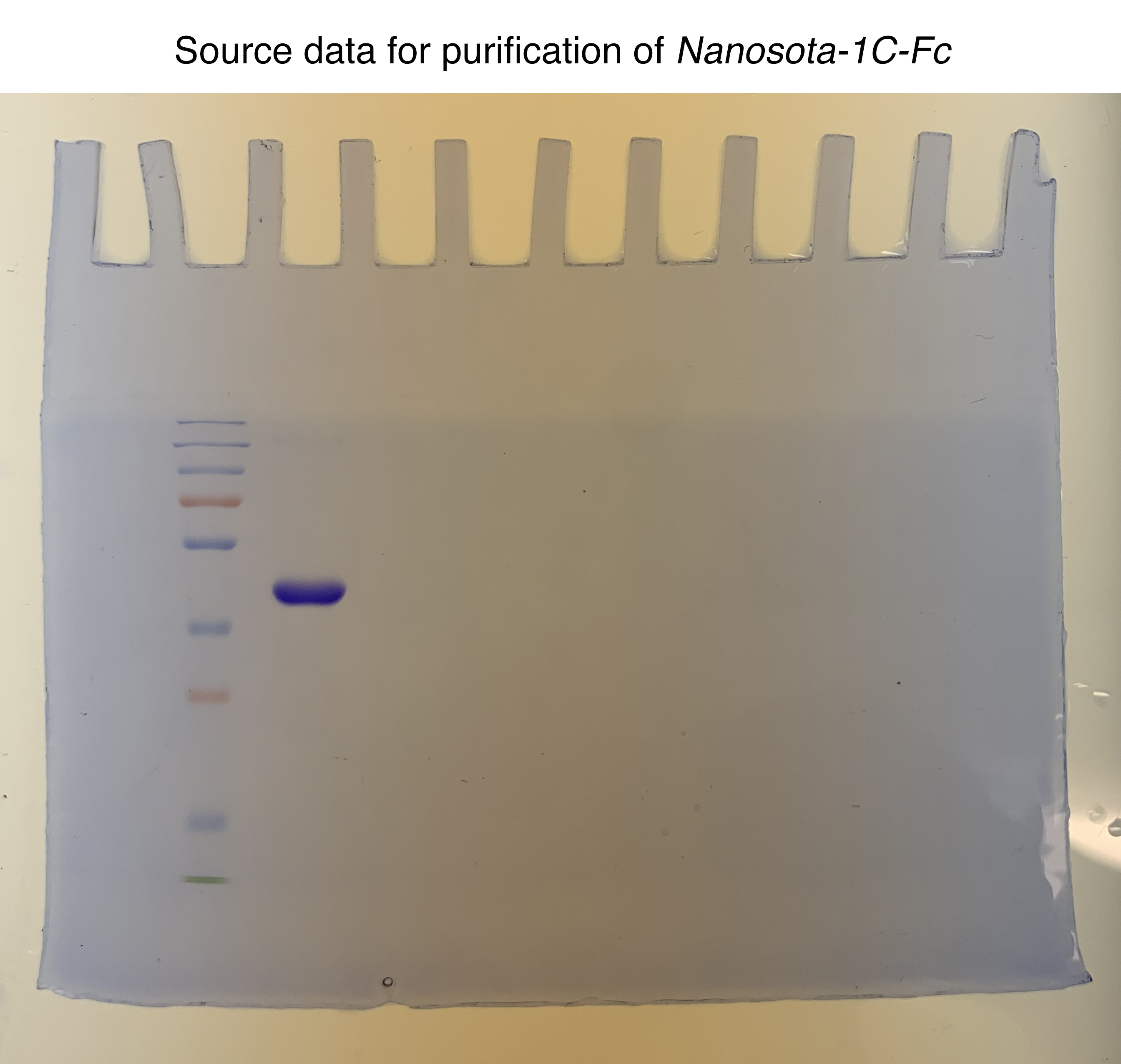

Supplement: Figure 5—source data 1. [file elife-64815-fig5-data1.zip › Figure 5A-source data 1/Figure 5A-source data 1.tif]

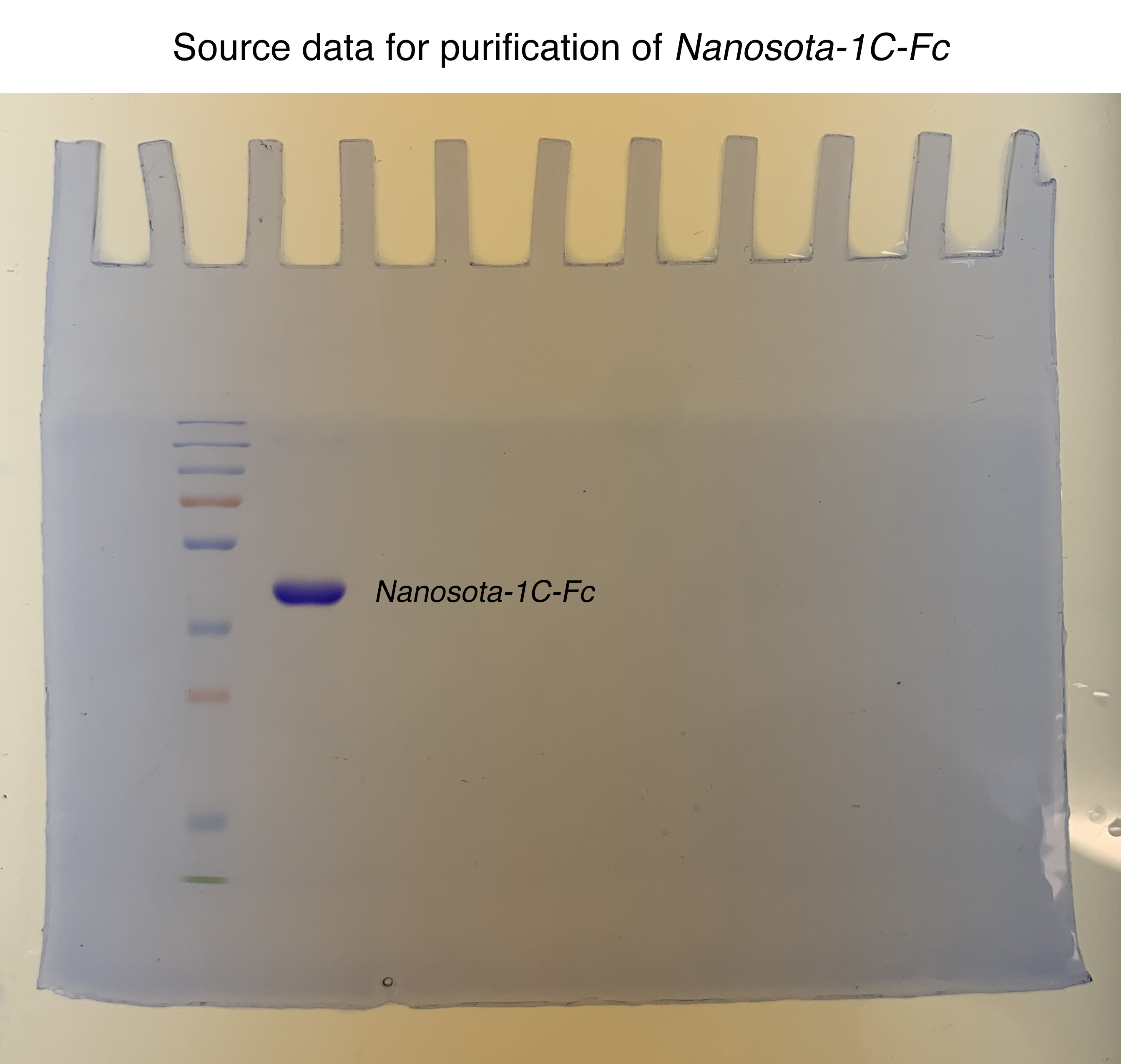

Supplement: Figure 5—source data 1. [file elife-64815-fig5-data1.zip › Figure 5A-source data 1/Figure 5A-source data 1 labeled.tif]
