# Supplementary material for: The development of Nanosota-1 as anti-SARS-CoV-2 nanobody drug candidates
Source: eLife. 2021 Aug 2;10:e64815. doi: 10.7554/eLife.64815 (PMC8354634; doi:10.7554/eLife.64815)
Supplement: Figure 4—source data 1. [file elife-64815-fig4-data1.zip › Figure 4D-source data 1/Mouse lung histology.docx]

**Source files for “Histology: lungs (mice)” (related to Figure 4D)**

This zip archive contains all original images shown in Figure 4D. The filename of each source data indicates which panel of the figure it is related to in the figure 4D. The ruler in each source data image indicates the magnification.
