# Supplementary material for: The development of Nanosota-1 as anti-SARS-CoV-2 nanobody drug candidates
Source: eLife. 2021 Aug 2;10:e64815. doi: 10.7554/eLife.64815 (PMC8354634; doi:10.7554/eLife.64815)
Supplement: Figure 5—source data 1. [file elife-64815-fig5-data1.zip › Figure 5A-source data 1/SDS-PAGE gel.docx]

**Source files for “Analysis of expression and purification of *Nanosota-1C*-*Fc*” (related to Figure 5A)**

This zip archive contains the gel shown in Figure 5A. Both the original file of the full raw unedited gel and the figure with the uncropped gel with the relevant band labelled are included.
